# Identifying directed links in large scale functional networks: application to brain fMRI

**DOI:** 10.1186/1471-2121-8-S1-S5

**Published:** 2007-07-10

**Authors:** Guillermo A Cecchi, A Ravishankar Rao, Maria V Centeno, Marwan Baliki, A Vania Apkarian, Dante R Chialvo

**Affiliations:** 1 Computational Biology Center, T.J. Watson IBM Research Center, Yorktwon Heights, New York, USA; 2 Dept. of Physiology, Feinberg School of Medicine, Northwestern University, Chicago, Illinois, USA

## Abstract

**Background:**

Biological experiments increasingly yield data representing large ensembles of interacting variables, making the application of advanced analytical tools a forbidding task. We present a method to extract networks of correlated activity, specifically from functional MRI data, such that: (a) network nodes represent voxels, and (b) the network links can be directed or undirected, representing temporal relationships between the nodes. The method provides a snapshot of the ongoing dynamics of the brain without sacrificing resolution, as the analysis is tractable even for very large numbers of voxels.

**Results:**

We find that, based on topological properties of the networks, the method provides enough information about the dynamics to discriminate between subtly different brain states. Moreover, the statistical regularities previously reported are qualitatively preserved, i.e. the resulting networks display scale-free and small-world topologies.

**Conclusion:**

Our method expands previous approaches to render large scale functional networks, and creates the basis for an extensive and -due to the presence of mixtures of directed and undirected links- richer motif analysis of functional relationships.

## Background

A growing number of biological experiments are producing datasets consisting of large numbers of interacting variables, from genomics to neural networks to eco-systems, giving rise to the nascent field of systems biology. The eminent challenge of this discipline is how to simplify the analysis of these high dimensional dynamical systems while retaining their relevant features. In particular, despite the rich, complex dynamics of the brain, and the highly interconnected and non-linear nature of its information processing capabilities, the bulk of the literature on brain imaging involves slight variations on the main theme: the identification of the degree of correlation between the activation of a local brain area and external markers. A variety of attempts have been made trying to go beyond this paradigm, including ICA, Volterra kernels and supervised classification techniques [1-4]; however, the applicability of these multi-variate methods has been restricted to networks with only a few hundred vertices.

Recently, a different approach was introduced [5,6], based on the analysis of the structure of pair-wise correlations between voxels in fMRI, embedding it in a graph structure whose nodes are the voxels and edges between them are defined by the covariance exceeding a threshold. With this relatively simple approach, we were able to demonstrate that the resulting networks have universal statistical topological properties, like scale-free connectivity and small-worldness [7], shared with other biological networks. Moreover, the spatial distribution of these networks is determined by the specific task the brain is engaged in, as opposed to capturing a task-independent network with little functional sensitivity. Similar ideas have been used to analyze and discriminate a variety of functional and dysfunctional brain states, and indeed have confirmed our initial results [8-10].

Another approach to capture the dynamics of complex systems is the causality analysis pioneered by Granger. The essence of this approach is to identify possible causal relationship between the variables of a system by analyzing how much the time course of one variable contributes to that of another one, based on an auto-regressive model. The method has been applied with success to neural data in the context of a few electro-physiological recordings, and to small numbers of brain areas represented by aggregates of several voxels [11,12]. One aspect of Granger's method and its derivatives that makes it superior to a simple covariance analysis is that it takes care of the transitivity and symmetry of the covariance: if a variable *A *determines the dynamical behavior of variable *B*, and *B *in its turn drives *C*, the covariance will find symmetric "links" *A *↔ *B*, *B *↔ *C *and *A *↔ *C*; similarly, if *A *drives *B *and *C *but the latter two do not influence each other, the covariance will still find B and C to be linked. Multivariate methods based on Granger's causality are able to break the symmetry and the transitivity, and therefore provide a more accurate description of the internal dynamics of the system. These methods have nevertheless several limitations arising from the need of a very large number of samples, as the auto-regressive model requires a matrix whose dimensions are (*M *× *T*)^2^, where *M *is the number of brain areas, and *T *the number of time units looking into the past [13,14].

In the present work, we try to circumvent both the limitations of covariance-based analysis and auto-regression-based causality analysis. We extend our previous findings by attempting to capture a larger signature of the dynamics of the brain by including directionality in the edges of the network, based on the concept of delayed covariance.

In our previous work, we defined a functional network by considering all functional voxels {*v*_*i*_} as possible nodes; their covariance determines whether a binary functional link (or edge) exists between them: *c*_*ij *_= ⟨(*v*_*i*_(*t*) - v¯
 MathType@MTEF@5@5@+=feaafiart1ev1aaatCvAUfKttLearuWrP9MDH5MBPbIqV92AaeXatLxBI9gBaebbnrfifHhDYfgasaacH8akY=wiFfYdH8Gipec8Eeeu0xXdbba9frFj0=OqFfea0dXdd9vqai=hGuQ8kuc9pgc9s8qqaq=dirpe0xb9q8qiLsFr0=vr0=vr0dc8meaabaqaciaacaGaaeqabaqabeGadaaakeaacuWG2bGDgaqeaaaa@2E39@_*i*_)(*v*_*j*_(*t*) - v¯
 MathType@MTEF@5@5@+=feaafiart1ev1aaatCvAUfKttLearuWrP9MDH5MBPbIqV92AaeXatLxBI9gBaebbnrfifHhDYfgasaacH8akY=wiFfYdH8Gipec8Eeeu0xXdbba9frFj0=OqFfea0dXdd9vqai=hGuQ8kuc9pgc9s8qqaq=dirpe0xb9q8qiLsFr0=vr0=vr0dc8meaabaqaciaacaGaaeqabaqabeGadaaakeaacuWG2bGDgaqeaaaa@2E39@_*j*_)σi−1σj−1
 MathType@MTEF@5@5@+=feaafiart1ev1aaatCvAUfKttLearuWrP9MDH5MBPbIqV92AaeXatLxBI9gBaebbnrfifHhDYfgasaacH8akY=wiFfYdH8Gipec8Eeeu0xXdbba9frFj0=OqFfea0dXdd9vqai=hGuQ8kuc9pgc9s8qqaq=dirpe0xb9q8qiLsFr0=vr0=vr0dc8meaabaqaciaacaGaaeqabaqabeGadaaakeaaiiGacqWFdpWCdaqhaaWcbaGaemyAaKgabaGaeyOeI0IaeGymaedaaOGae83Wdm3aa0baaSqaaiabdQgaQbqaaiabgkHiTiabigdaXaaaaaa@370A@, where v¯
 MathType@MTEF@5@5@+=feaafiart1ev1aaatCvAUfKttLearuWrP9MDH5MBPbIqV92AaeXatLxBI9gBaebbnrfifHhDYfgasaacH8akY=wiFfYdH8Gipec8Eeeu0xXdbba9frFj0=OqFfea0dXdd9vqai=hGuQ8kuc9pgc9s8qqaq=dirpe0xb9q8qiLsFr0=vr0=vr0dc8meaabaqaciaacaGaaeqabaqabeGadaaakeaacuWG2bGDgaqeaaaa@2E39@_*i *_= ⟨(*v*_*i*_(*t*)⟩_*t *_and σi2
 MathType@MTEF@5@5@+=feaafiart1ev1aaatCvAUfKttLearuWrP9MDH5MBPbIqV92AaeXatLxBI9gBaebbnrfifHhDYfgasaacH8akY=wiFfYdH8Gipec8Eeeu0xXdbba9frFj0=OqFfea0dXdd9vqai=hGuQ8kuc9pgc9s8qqaq=dirpe0xb9q8qiLsFr0=vr0=vr0dc8meaabaqaciaacaGaaeqabaqabeGadaaakeaaiiGacqWFdpWCdaqhaaWcbaGaemyAaKgabaGaeGOmaidaaaaa@30F0@ = ⟨(*v*_*i*_(*t*) - v¯
 MathType@MTEF@5@5@+=feaafiart1ev1aaatCvAUfKttLearuWrP9MDH5MBPbIqV92AaeXatLxBI9gBaebbnrfifHhDYfgasaacH8akY=wiFfYdH8Gipec8Eeeu0xXdbba9frFj0=OqFfea0dXdd9vqai=hGuQ8kuc9pgc9s8qqaq=dirpe0xb9q8qiLsFr0=vr0=vr0dc8meaabaqaciaacaGaaeqabaqabeGadaaakeaacuWG2bGDgaqeaaaa@2E39@_*i*_)^2^⟩, such that if the correlation between *i *and *j *exceeds a threshold, a functional link is considered, and none otherwise: **if ***c*_*ij *_> *C*_*T *_**then ***d*_*ij *_= 1, **else ***d*_*ij *_= 0. We extend here this approach by considering the delayed or lagged covariance: *c*_*ij*_(*τ*) = ⟨(*v*_*i*_(*t *- *τ*)- v¯
 MathType@MTEF@5@5@+=feaafiart1ev1aaatCvAUfKttLearuWrP9MDH5MBPbIqV92AaeXatLxBI9gBaebbnrfifHhDYfgasaacH8akY=wiFfYdH8Gipec8Eeeu0xXdbba9frFj0=OqFfea0dXdd9vqai=hGuQ8kuc9pgc9s8qqaq=dirpe0xb9q8qiLsFr0=vr0=vr0dc8meaabaqaciaacaGaaeqabaqabeGadaaakeaacuWG2bGDgaqeaaaa@2E39@_*i*_)(*v*_*j*_(*t*) - v¯
 MathType@MTEF@5@5@+=feaafiart1ev1aaatCvAUfKttLearuWrP9MDH5MBPbIqV92AaeXatLxBI9gBaebbnrfifHhDYfgasaacH8akY=wiFfYdH8Gipec8Eeeu0xXdbba9frFj0=OqFfea0dXdd9vqai=hGuQ8kuc9pgc9s8qqaq=dirpe0xb9q8qiLsFr0=vr0=vr0dc8meaabaqaciaacaGaaeqabaqabeGadaaakeaacuWG2bGDgaqeaaaa@2E39@_*j*_)σi−1σj−1
 MathType@MTEF@5@5@+=feaafiart1ev1aaatCvAUfKttLearuWrP9MDH5MBPbIqV92AaeXatLxBI9gBaebbnrfifHhDYfgasaacH8akY=wiFfYdH8Gipec8Eeeu0xXdbba9frFj0=OqFfea0dXdd9vqai=hGuQ8kuc9pgc9s8qqaq=dirpe0xb9q8qiLsFr0=vr0=vr0dc8meaabaqaciaacaGaaeqabaqabeGadaaakeaaiiGacqWFdpWCdaqhaaWcbaGaemyAaKgabaGaeyOeI0IaeGymaedaaOGae83Wdm3aa0baaSqaaiabdQgaQbqaaiabgkHiTiabigdaXaaaaaa@370A@. We reason as follows: if there is a significant peak of the covariance between *i *and *j *at zero lag, then there is a potential binary symmetric link between them, as before. However, if the significant peak is not at zero lag, then we will consider that the preceding voxel, and only it, has a directed link pointing to the succeeding one. That is, **if ***c*_*ij*_(*τ *= 0) > *C*_*T *_**then ***d*_*ij *_= *d*_*ji *_= 1; **else if ***c*_*ij*_(*τ *> 0) > *C*_*T *_**then ***d*_*ij *_= 1 **and ***d*_*ji *_= 0; **else ***d*_*ij *_= 0.

In other words, two voxels whose activity is highly correlated and simultaneous are considered to be symmetrically linked; a voxel that is highly correlated with the future of another one will be considered as a "source", and the latter as a "sink". This approach clearly can break the symmetry of the covariance, but as described cannot deal with the problem of the transitivity described above. Taking into consideration the relatively poor temporal resolution of fMRI, we reasoned that for the time being we could only in earnest tackle one of the confounding sources of undirected links, namely the explanation of a zero-lag covariance (i.e. undirected link) between two voxels by the presence of a common source, of which they are both targets. That is, after identifying all sources and sinks, every potential undirected link that can be explained by a common source is removed. We also considered possible reductions of triangulations of directed links (as in *A *→ *B*, *B *→ *C*, *A *→ *C*), but we did not find this approach to have a significant impact, presumably due to the low temporal resolution of the signal (data not shown, see the Methods section).

## Results

We analyzed a dataset discussed in the literature [6], consisting of subjects undertaking a simple self-paced finger-tapping task, for the purpose of exploring the potential of our method. The task is composed of three variants, which were designed to be relatively similar, in the hope that the method could discriminate subtle differences in the patterns of brain activation. The variation between the tasks is based on the cue utilized to signal the start and stop of the finger-tapping. In the first task, a small featureless visual cue presented in the center of the screen; in the second task the cue is a short auditory tone; in the third one a visual cue similar to the first one is presented, the only difference being its larger size.

After standard functional pre-processing (see Methods), and delayed covariance analysis as explained above, the resulting networks were studied using the tools of statistical network theory. The first observation is that most links are undirected, comprising on average of 50% to 60% of total links; this is compatible with the notion that most neural interactions result from fast, local and presumably symmetric connections, whose subtle dynamics are for the most part beyond the present reach of functional MRI. However, directed links account for a significant number of the observed correlations between voxels, suggesting that our approach can indeed be fruitful in terms of capturing ongoing dynamics.

**Figure 1:**
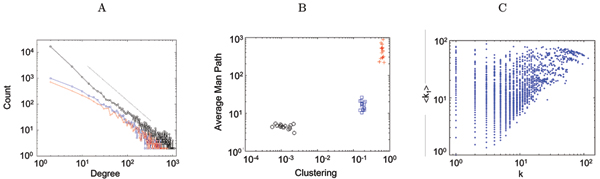
(A) Degree distribution of links. The black trace corresponds to the degree for all the links (directed and undirected), the blue one to the degree of sources (out-directed links) and the red trace to sinks (in-directed links), averaged over subjects. Observe that (a) both directed link degree distributions are very similar, and (b) there is a scale-free trend, more marked when all links are considered. The dotted line corresponds to a power-law of 3/2. (B) Small-world topology of the networks. Clustering and average minimal path for functional networks (open circles), equivalent Erdös random networks (red crosses) and equivalent regular lattices (blue x's). Data points correspond to different subjects and tasks. (C) Assortative mixing of directed networks. The horizontal axis represents the total degree of each node, and the vertical axis the average degree of the first neighbors; all the data points correspond to one single subject.

Although directed links have less statistical power than undirected ones, their degree distribution still shows a power-law behavior. This is exemplified in Fig. 1A, where the histogram of source (blue) and sink (red) degrees, i.e. out- and in-directed links, averaged over all subjects and tasks, is represented. Observe that the distribution is very similar for both classes of links, and approaches a power-law with an exponent of 3/2, which is even more evident when all links are considered for each node (black). This result is in line with our previous findings, as it reinforces the notion of information hubs dominating the flow of information in the brain. The effect of removing some of the undirected edges is to reduce the scaling from an exponent near 2 as estimated with our previous approach. Figure 1B depicts the result of analyzing the small-world topology of the functional networks. The panel represents the clustering vs. average minimal path for all the studied cases (open circles), the equivalent random networks (red crosses), and the equivalent regular lattices (blue x's). Observe that the clustering of the functional networks is several orders of magnitude larger than that of the equivalent random networks, and comparable to that of the equivalent regular lattices. The average minimal path of the functional networks, while larger than that of the random networks, is still smaller than that of the equivalent regular lattices. In comparison with our previous study, the main topological features are preserved: for the same threshold (0.7), there is an increase from 12.9 to 16.3 in the average path due to the removal of undirected links explained by a common source; interestingly, the clustering coefficient increases from 0.12 to 0.18, presumably due to triangulations that could not be detected before. Similarly, a comparison with our previous finding regarding the assortative nature of functional brain networks shows that indeed this property does not change when directed links are included. Figure 1C represents the total degree of each node vs. the average degree of the first neighbors, i.e. nodes reachable through in-, out- and undirected edges. Again, we find a correlation between these two degrees, in contrast with all other biological networks investigated to date, which implies a lack of hierarchical organization in the networks: a hierarchical network would display a negative correlation, such that nodes with high degree (or hub nodes) tend to be connected with low degree nodes (or peripheral nodes). A more detailed study, analyzing differences between in- and out-hubs, will be reported in future publications.

**Figure 2:**
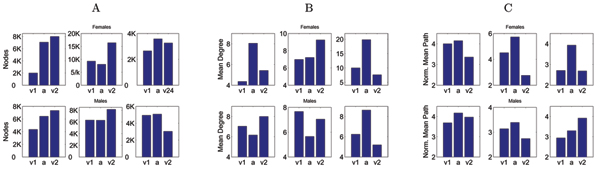
Discrimination of tasks based on global topological properties. The first column of data points corresponds to the small visual cue task, the second to the auditory cue, and the third to the large visual cue in all panels. The upper row corresponds to female subjects, and the lower one to males. (A) Total number of nodes. (B) Mean degree of the networks. (C) Normalized mean path for each network. Observe the inverted V shape in 5 out of the 6 subjects, such that the normalized mean path of the auditory cue task is consistently larger than that of both visual cue tasks.

Interestingly, the networks hold enough information about the dynamics of brain states so that even a global measure of their properties can discriminate between tasks. Figure 2 shows the result of comparing different topological measures of the three resulting networks (small visual cue, auditory cue, large visual cue tasks) for 6 different subjects. The first observation in this regard is that the total number of nodes identified by the method (Panel 2A) is not a good indicator of the identity of the task, or of the subject for that matter; this is to be expected, as the number of nodes depends dramatically on the chosen threshold. The average degree, or connectivity (Panel 2B), does not seem to provide much information either; this is clearly a very local measure of the structure of the network, for the most part unrelated to the flow of information. We observed a tendency towards discriminating between the tasks when we computed the average minimal path of the networks (not shown), defined as the minimal number of links needed to reach a node from another one, averaged over all nodes. This tendency, though, is not consistent across subjects, as the auditory cue task is in some cases large and small in others. However, we were able to improve this measurement by considering the normalized mean path, as the mean path of the network over that of the equivalent Erdös random network, displayed in Panel 2C (see Methods). Observe that for 5 out of the 6 subjects, the auditory task is consistently larger (i.e. has a bigger normalized mean path) that the equivalent visual cue tasks. Non-parametric statistical analysis on the rank of the normalized mean path (ℒ
 MathType@MTEF@5@5@+=feaafiart1ev1aaatCvAUfKttLearuWrP9MDH5MBPbIqV92AaeXatLxBI9gBaebbnrfifHhDYfgasaacH8akY=wiFfYdH8Gipec8Eeeu0xXdbba9frFj0=OqFfea0dXdd9vqai=hGuQ8kuc9pgc9s8qqaq=dirpe0xb9q8qiLsFr0=vr0=vr0dc8meaabaqaciaacaGaaeqabaqabeGadaaakeaat0uy0HwzTfgDPnwy1egaryqtHrhAL1wy0L2yHvdaiqaacqWFsectaaa@376D@_*a *_> ℒ
 MathType@MTEF@5@5@+=feaafiart1ev1aaatCvAUfKttLearuWrP9MDH5MBPbIqV92AaeXatLxBI9gBaebbnrfifHhDYfgasaacH8akY=wiFfYdH8Gipec8Eeeu0xXdbba9frFj0=OqFfea0dXdd9vqai=hGuQ8kuc9pgc9s8qqaq=dirpe0xb9q8qiLsFr0=vr0=vr0dc8meaabaqaciaacaGaaeqabaqabeGadaaakeaat0uy0HwzTfgDPnwy1egaryqtHrhAL1wy0L2yHvdaiqaacqWFsectaaa@376D@_*sv *_and ℒ
 MathType@MTEF@5@5@+=feaafiart1ev1aaatCvAUfKttLearuWrP9MDH5MBPbIqV92AaeXatLxBI9gBaebbnrfifHhDYfgasaacH8akY=wiFfYdH8Gipec8Eeeu0xXdbba9frFj0=OqFfea0dXdd9vqai=hGuQ8kuc9pgc9s8qqaq=dirpe0xb9q8qiLsFr0=vr0=vr0dc8meaabaqaciaacaGaaeqabaqabeGadaaakeaat0uy0HwzTfgDPnwy1egaryqtHrhAL1wy0L2yHvdaiqaacqWFsectaaa@376D@_*a *_> ℒ
 MathType@MTEF@5@5@+=feaafiart1ev1aaatCvAUfKttLearuWrP9MDH5MBPbIqV92AaeXatLxBI9gBaebbnrfifHhDYfgasaacH8akY=wiFfYdH8Gipec8Eeeu0xXdbba9frFj0=OqFfea0dXdd9vqai=hGuQ8kuc9pgc9s8qqaq=dirpe0xb9q8qiLsFr0=vr0=vr0dc8meaabaqaciaacaGaaeqabaqabeGadaaakeaat0uy0HwzTfgDPnwy1egaryqtHrhAL1wy0L2yHvdaiqaacqWFsectaaa@376D@_*lv*_) yields a p-value of 0.017. This indicates that the subtle differences in activation elicited by the tasks have a measurable effect in the overall structure of correlations and flow of information of the networks. This tendency was probably amplified by the fact that only the *giant *component of the network was considered for the purpose of measuring the average minimal path. The giant component is defined as the largest connected sub-network of a graph; we observed that in all cases this component was at least one order of magnitude larger in size that the runner-ups. In other words, we targeted a *core *of correlated activity and disregarded much smaller sub-networks that could otherwise be relevant. It is also worth mentioning that the directional nature of the links enters explicitly the computation of the mean path, as a link *A *→ *B *means that there is no direct access from *B *to *A*.

**Figure 3:**
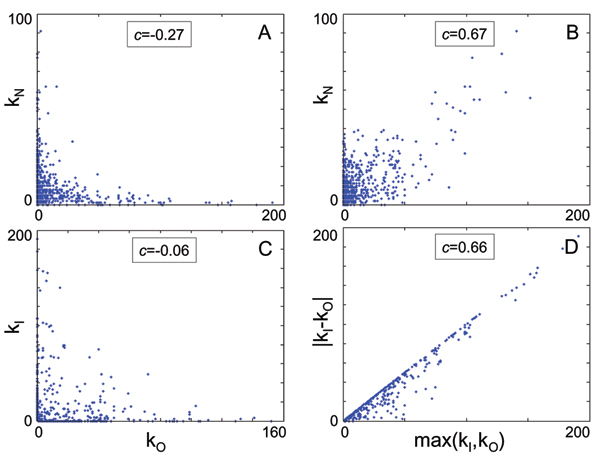
Panel A: relationship between the in-degree and the undirected-degree for each node; observe that there is a negative correlation, i.e. in-hubs tend to have very few undirected links and vice versa (insets correspond to covariance between the plotted variables). Panel B: same as Panel A, but for the out-degree of the nodes; in this case, there is a strong correlation between out-hubs and undirected-hubs. Panel C: relationship between out- and in-degree for each node. The plot makes evident that nodes tend to be either in-hubs or out-hubs, as the correlation between the degrees is basically insignificant; moreover, large hubs tend to lay on the axes, i.e. they have a bias to be pure "sources" (horizontal) or "sinks" (vertical). Another way to see this phenomenon is presented in Panel D, where the maximum between the in- and out-degrees is plotted against the absolute value of the difference between the same quantities. As the plot shows, nodes tend to cluster near the identity line, which corresponds to pure sources and sinks.

A remarkable regularity displayed by these networks is the tendency for nodes to be mostly "sources" (i.e. heavy out-hubs) or "sinks" (heavy in-hubs). That is, nodes with a large number of out-links tend to have relatively few in-edges, and vice versa, although, interestingly, this is not a strictly enforced rule. Moreover, in-hubs tend to have relatively few undirected links, whereas out-hubs tend to be also undirected hubs. This seems to be counter-intuitive at face value, as one may naïvely think that the hubs are balanced; however, they need not be so, as one would expect in, for instance, tracffic hubs. In other words, there are no conserved quantities at the hub level to be balanced. These results are summarized in Fig. 3. The tendency for nodes to be either in- or out-hubs is particularly clear in Panel D, where the maximum between the in-degree and the out-degree is plotted against the absolute value of their difference, showing a strong correlation.

## Discussion

Topological regularities of functional brain networks have been described before in the literature, including our own work, but they were based on a narrower window on the properties of the underlying dynamical system (i.e. zero-lag correlation), and could not provide for discernibility of subtly different brain states. The finding that hybrid networks with directed and undirected links can be discriminated based on global topological measures is very relevant to theoretical approaches to brain function, as it is a formalization of the collective properties of complex systems.

**Figure 4:**
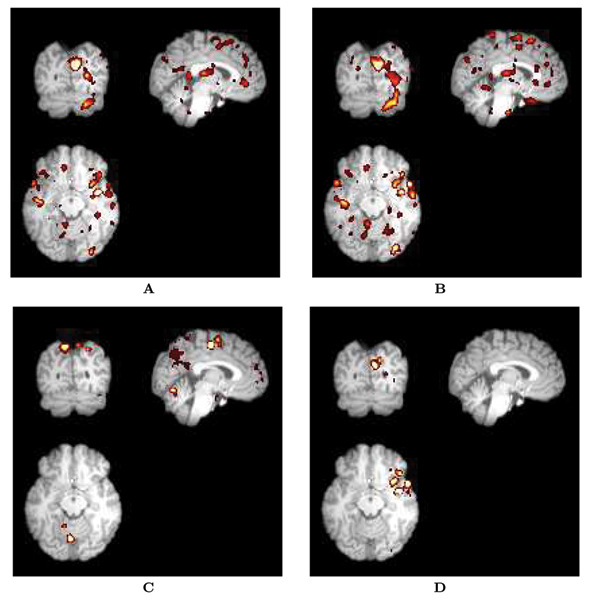
Density maps of undirected and directed links. The local degree of neutral or undirected connectivity is color-coded, such that high brightness signifies high degree. (A) Undirected links, small visual cue. (B) Undirected links, auditory cue. (C) Directed links, small visual cue. (D) Directed links, auditory cue. Observe the similarity between the undirected links maps for the two different tasks (A and B), while the directed links maps are remarkably different (C and D).

However, to move beyond a simply phenomenological description of such systems, we are compelled to bridge the gap between emergent global behavior and local functional properties. One possibility is to interpret the results depicted in Fig. 2C in terms of the spatial organization of the networks. To do so, and given the limitations of representing the full complexity of the networks, we resorted to a simplified approach that captures only part of this complexity but serves as a possible guide for reasoning. Figure 4 represents the networks by link density maps, where the nodes are depicted in their original spatial location, and the links are mapped using a color-coded representation of the degree (or number of connections). The degree of undirected links is shown for the same subject in the small visual task in Panel A, and the auditory cue task in Panel B. Observe that the density maps are very similar in both conditions. The out-degree, i.e. sources of directed links, is depicted in Panels C for the visual cue task and Panel D for auditory. In this case, the differences between the maps are much more compelling; however, it is to be expected that precisely in a class of tasks where the varying factor is a short cue, the main source of functional differences will be "sources of information", in a manner of speak. A thorough analysis of the regional discernibility of the method is beyond the scope of the present publication, focused on topological properties of the networks. This would require a systematic topographic comparison of topological features (such as degree distribution) across subjects and tasks, beyond the scope of this manuscript.

The other novel, and certainly unexpected, result is the one summarized in Figure 3: the nodes of the networks tend to be either in- or out-hubs of directed links. The interpretation of this finding is not straightforward; at the neuronal level, we would expect a more balanced relation between "driving" and "driven" connections subtending hypothetical closed circuits across the brain during the completion of a task. However, this interpretation cannot proceed at the level of resolution that the technique provides. In purely speculative terms, this finding points to a role for very large neuronal patches, possibly entire functional areas, in driving other large patches at a relatively slow time scale, compatible with that of the task. Under this preliminary interpretation, the observed in-hub/out-hub exclusion would be an emergent feature of neuronal ensembles, amplified by the highly non-linear (i.e. binary) process of rendering directed links. We are currently studying different alternatives to clarify these ideas, most prominently ensemble modeling.

## Conclusion

Building on our previous work on graph theoretic analysis of functional magnetic resonance imaging data, we have introduced a novel method to capture more of the complex dynamical interactions of brain networks. We find that this approach yields networks with similar topological properties as those described previously, i.e. they display scale-free connectivity, non-hierarchical architecture and small-world topology, even when considering only the giant component for the analysis and strictly enforcing directionality in the computation of the average mean path. However, the topological information contained in directed and undirected links is enough to reveal subtle brain state differences, consisting of a very short auditory or visual cue to trigger a relatively much longer finger-tapping motor sequence. Initial results suggest that these topological differences are concurrent with distinct patterns in the spatial distribution of hubs of directed links, i.e. the location of in- and out-hubs. These findings point in the direction of a functional dissection based on the density and architecture of directed connections, and more specifically on the spatial patterns of topological motifs, similar to approaches already advanced in the study of genetic networks [15,16]. This is the subject of current investigation in our group.

Finally, we conclude that the computational feasibility of the approach (see Methods), even when dealing with 10,000 to 20,000 independent variables, renders it applicable to other similarly large biological networks, like gene-expression patterns generated by cDNA microarrays [17].

## Methods

**Figure 5:**
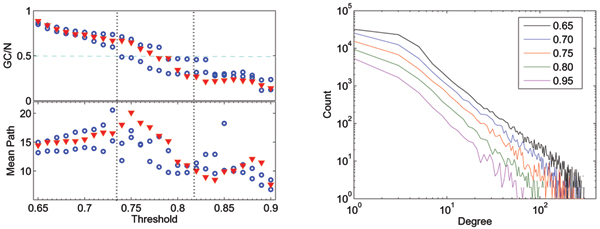
Upper left: relative size of the giant connected component (*GC*) respect to the whole network (*N*), as a function of the threshold. Lower left: mean path of the giant component as a function of the threshold. In both cases, circles represent visual cue task, and triangles auditory cue task networks. Right panel: degree distribution as a function of the threshold. Each color represents the distribution obtained with a different threshold, for for the same subject and task; observe the slight difference in the exponent respect to the average in Fig. 1A.

**Figure 6:**
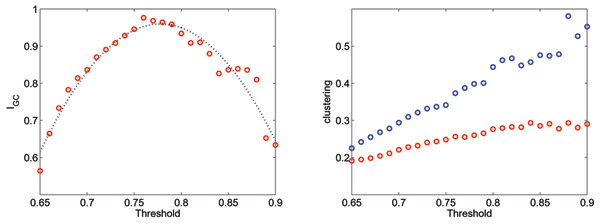
Left: distance of the relative size of the giant connected component to 50%, averaged over different networks. This emphasizes that the giant component tends to be near 50% for the chosen threshold values. Right: change of the clustering for the giant component (blue) and the entire network (red).

The implementation of the delayed covariance calculation is relatively straight-forward, although it can be challenging from a computational-resources point-of-view. In a typical functional task, there are in the order of 20,000 voxels with significant zero-lag covariance, for functional scans with a resolution of 64 × 64 × 36; for each of them we need to compute a delayed covariance. This was implemented in a 24-way shared-memory machine in an MPI environment, taking approx. 2 hrs. for scans of 400 volumes and a time window of 11 time points; the same algorithm, also in MPI, takes 8 minutes in a 1024-way IBM Blue Gene rack [18]. The weeding of confounding undirected links, although not altogether simple from an algorithmic point-of-view, runs extremely fast even in a single processor machine. The weeding process results, on average, in the weeding out of 15–20% of potential undirected links, and the final networks have an average connectivity (including directed and undirected links) of approx. 15 to 20 links per node. In all cases, the threshold for zero-lag covariance to determine a link was uniformly set to 0.75, whereas that for non-zero-lag covariance was set to 0.7, under the assumption that the various noise factors and the limited temporal sampling affect this measure more drastically. Whereas we cannot provide an analytic criterion for choosing the threshold, the selection was based on the following rationale: firstly, the threshold should not be so low that the giant component encompasses the whole brain, as we expect to capture the core of the activity; secondly, it should not be so high as to encompass only the most active elements, which would likely be identified by linear methods. In fact, we found that a reasonable criterion is for the giant component to include around 50% of all the nodes in the network; this typically occurs within a range of thresholds that includes the one we have selected, as illustrated in Fig. 5. The upper left panel shows the relative size of the giant component as a function of the threshold, for two visual cue tasks (circles) and auditory cue task (triangles) in one subject. All the networks cross the 50% mark in a threshold interval between 0.73 and 0.82; correspondingly, this is the interval within which the discrimination of the tasks is most accurate, as represented by the change of the mean path as a function of the threshold, in the lower left panel of the same figure. Further reassuring the robustness of the method, the right panel represents the change of the degree distribution with the threshold. Similarly to what was previously reported [6] for networks based only on zero-lag covariance, the scaling is very stable over a wide range of thresholds; it is to be noted that this measured on the entire network, as opposed to the giant component. This robustness is also highlighted by the fact that the clustering index, even though it changes as a function of the threshold, remains significantly above what would be expected from a random network, as depicted in Fig. 6, right panel (blue circles); the average over the entire network is even less sensitive (red). Finally, the left panel of Fig. 6 shows a measure of how close the giant component is to encompass half of the nodes of the entire network: the plot represents a unimodal function of the ratio *p *= *GC/N *that peaks at 1/2, *I*_*GC *_= *p *log_2 _*p *+ (1 -*p*) log_2_(1 -*p*), averaged over networks. Observe again that *I*_*GC *_is close to the maximum of 1 for a range of thresholds that includes the one we selected. In all cases, the threshold for non-zero-lag was set 0.05 below that for zero-lag, for the reasons explained above.

The weeding of confounding undirected or neutral links was implemented as follows: if two nodes have an undirected link, *A *↔ *B*, but they have a common directed source, *A *→ *B *and *A *→ *C*, then the undirected link is removed. We considered directed triangulations with commensurable time delays: *A *→ *B*, *B *→ *C*, *A *→ *C *and Δ*τ*(*A*, *B*) + Δ*τ*(*B*, *C*) = Δ*τ*(*A*, *C*), such that the links *B *→ *C *or *A *→ *C *could be confounds. The statistical significance of these configurations was very low, and therefore we decided not to include them in the present analysis. The average minimal path or geodesic of a graph is defined as the average over all nodes of the shortest distance from one node to every other ℒ
 MathType@MTEF@5@5@+=feaafiart1ev1aaatCvAUfKttLearuWrP9MDH5MBPbIqV92AaeXatLxBI9gBaebbnrfifHhDYfgasaacH8akY=wiFfYdH8Gipec8Eeeu0xXdbba9frFj0=OqFfea0dXdd9vqai=hGuQ8kuc9pgc9s8qqaq=dirpe0xb9q8qiLsFr0=vr0=vr0dc8meaabaqaciaacaGaaeqabaqabeGadaaakeaat0uy0HwzTfgDPnwy1egaryqtHrhAL1wy0L2yHvdaiqaacqWFsectaaa@376D@ = ⟨ℒ
 MathType@MTEF@5@5@+=feaafiart1ev1aaatCvAUfKttLearuWrP9MDH5MBPbIqV92AaeXatLxBI9gBaebbnrfifHhDYfgasaacH8akY=wiFfYdH8Gipec8Eeeu0xXdbba9frFj0=OqFfea0dXdd9vqai=hGuQ8kuc9pgc9s8qqaq=dirpe0xb9q8qiLsFr0=vr0=vr0dc8meaabaqaciaacaGaaeqabaqabeGadaaakeaat0uy0HwzTfgDPnwy1egaryqtHrhAL1wy0L2yHvdaiqaacqWFsectaaa@376D@_*g*_(*i*, *j*)⟩, and it has a simple interpretation as the "ease" of navigation of the network. The p-value for the data in Fig. 2C was computed as *p *= ∏(6, 5)*q*^5^(1 - *q*) + ∏(6, 6)*q*^6^, where ∏(*a*, *b*) is the combinatorial number *a*!/*b*!(*a *- *b*)! and *q *is the probability for the normalized mean path of the auditory cue network to be bigger than that of the small and large visual cues, for the null hypothesis: *q *= *P*(ℒ
 MathType@MTEF@5@5@+=feaafiart1ev1aaatCvAUfKttLearuWrP9MDH5MBPbIqV92AaeXatLxBI9gBaebbnrfifHhDYfgasaacH8akY=wiFfYdH8Gipec8Eeeu0xXdbba9frFj0=OqFfea0dXdd9vqai=hGuQ8kuc9pgc9s8qqaq=dirpe0xb9q8qiLsFr0=vr0=vr0dc8meaabaqaciaacaGaaeqabaqabeGadaaakeaat0uy0HwzTfgDPnwy1egaryqtHrhAL1wy0L2yHvdaiqaacqWFsectaaa@376D@_*a *_> ℒ
 MathType@MTEF@5@5@+=feaafiart1ev1aaatCvAUfKttLearuWrP9MDH5MBPbIqV92AaeXatLxBI9gBaebbnrfifHhDYfgasaacH8akY=wiFfYdH8Gipec8Eeeu0xXdbba9frFj0=OqFfea0dXdd9vqai=hGuQ8kuc9pgc9s8qqaq=dirpe0xb9q8qiLsFr0=vr0=vr0dc8meaabaqaciaacaGaaeqabaqabeGadaaakeaat0uy0HwzTfgDPnwy1egaryqtHrhAL1wy0L2yHvdaiqaacqWFsectaaa@376D@_*sv*_, ℒ
 MathType@MTEF@5@5@+=feaafiart1ev1aaatCvAUfKttLearuWrP9MDH5MBPbIqV92AaeXatLxBI9gBaebbnrfifHhDYfgasaacH8akY=wiFfYdH8Gipec8Eeeu0xXdbba9frFj0=OqFfea0dXdd9vqai=hGuQ8kuc9pgc9s8qqaq=dirpe0xb9q8qiLsFr0=vr0=vr0dc8meaabaqaciaacaGaaeqabaqabeGadaaakeaat0uy0HwzTfgDPnwy1egaryqtHrhAL1wy0L2yHvdaiqaacqWFsectaaa@376D@_*a *_> ℒ
 MathType@MTEF@5@5@+=feaafiart1ev1aaatCvAUfKttLearuWrP9MDH5MBPbIqV92AaeXatLxBI9gBaebbnrfifHhDYfgasaacH8akY=wiFfYdH8Gipec8Eeeu0xXdbba9frFj0=OqFfea0dXdd9vqai=hGuQ8kuc9pgc9s8qqaq=dirpe0xb9q8qiLsFr0=vr0=vr0dc8meaabaqaciaacaGaaeqabaqabeGadaaakeaat0uy0HwzTfgDPnwy1egaryqtHrhAL1wy0L2yHvdaiqaacqWFsectaaa@376D@_*lv*_) = 1/3. The clustering is defined as the average of the ratio, for each node, between how many of its neighbors share connections amongst them, and the total possible number of connections C
 MathType@MTEF@5@5@+=feaafiart1ev1aaatCvAUfKttLearuWrP9MDH5MBPbIqV92AaeXatLxBI9gBamrtHrhAL1wy0L2yHvtyaeHbnfgDOvwBHrxAJfwnaebbnrfifHhDYfgasaacH8akY=wiFfYdH8Gipec8Eeeu0xXdbba9frFj0=OqFfea0dXdd9vqai=hGuQ8kuc9pgc9s8qqaq=dirpe0xb9q8qiLsFr0=vr0=vr0dc8meaabaqaciaacaGaaeqabaWaaeGaeaaakeaaimaacqWFce=qaaa@3825@ = ⟨*N*_*i*_/∏(*k*_*i*_, 2)⟩, where *k*_*i *_is the number of neighbors of node *i*, and *N*_*i *_is the number of connections between the neighbors. This topological measure can also be interpreted as the density of triangulations of the network, as reflecting the presence of local structures. The theory developed by Erdös establishes that the geodesic path and clustering of a random network with *N *vertices and average connectivity *k *are, respectively: ℒ
 MathType@MTEF@5@5@+=feaafiart1ev1aaatCvAUfKttLearuWrP9MDH5MBPbIqV92AaeXatLxBI9gBaebbnrfifHhDYfgasaacH8akY=wiFfYdH8Gipec8Eeeu0xXdbba9frFj0=OqFfea0dXdd9vqai=hGuQ8kuc9pgc9s8qqaq=dirpe0xb9q8qiLsFr0=vr0=vr0dc8meaabaqaciaacaGaaeqabaqabeGadaaakeaat0uy0HwzTfgDPnwy1egaryqtHrhAL1wy0L2yHvdaiqaacqWFsectaaa@376D@ = log(*N*)/log(*k*), C
 MathType@MTEF@5@5@+=feaafiart1ev1aaatCvAUfKttLearuWrP9MDH5MBPbIqV92AaeXatLxBI9gBamrtHrhAL1wy0L2yHvtyaeHbnfgDOvwBHrxAJfwnaebbnrfifHhDYfgasaacH8akY=wiFfYdH8Gipec8Eeeu0xXdbba9frFj0=OqFfea0dXdd9vqai=hGuQ8kuc9pgc9s8qqaq=dirpe0xb9q8qiLsFr0=vr0=vr0dc8meaabaqaciaacaGaaeqabaWaaeGaeaaakeaaimaacqWFce=qaaa@3825@ = *k*/*N*. The alternative null hypothesis is a regular lattice. The mean geodesic path and clustering for the regular 1d-lattice in the original model proposed by Strogatz and Watts can also be computed as ℒ
 MathType@MTEF@5@5@+=feaafiart1ev1aaatCvAUfKttLearuWrP9MDH5MBPbIqV92AaeXatLxBI9gBaebbnrfifHhDYfgasaacH8akY=wiFfYdH8Gipec8Eeeu0xXdbba9frFj0=OqFfea0dXdd9vqai=hGuQ8kuc9pgc9s8qqaq=dirpe0xb9q8qiLsFr0=vr0=vr0dc8meaabaqaciaacaGaaeqabaqabeGadaaakeaat0uy0HwzTfgDPnwy1egaryqtHrhAL1wy0L2yHvdaiqaacqWFsectaaa@376D@ = *N*/2*k *and C
 MathType@MTEF@5@5@+=feaafiart1ev1aaatCvAUfKttLearuWrP9MDH5MBPbIqV92AaeXatLxBI9gBamrtHrhAL1wy0L2yHvtyaeHbnfgDOvwBHrxAJfwnaebbnrfifHhDYfgasaacH8akY=wiFfYdH8Gipec8Eeeu0xXdbba9frFj0=OqFfea0dXdd9vqai=hGuQ8kuc9pgc9s8qqaq=dirpe0xb9q8qiLsFr0=vr0=vr0dc8meaabaqaciaacaGaaeqabaWaaeGaeaaakeaaimaacqWFce=qaaa@3825@ = 3(*k *- 1)/4(*k *- 2). Erdös random networks are constructed so that an edge can be attached to any two nodes with equal a priori probability, and display a binomial degree distribution, low clustering and small mean path. For the purpose of our analysis, we compared the functional networks with Erdös networks with the same number of nodes and edges. The covariance in Fig. 3 is defined as *c*_*xy *_= ⟨(*x *- x¯
 MathType@MTEF@5@5@+=feaafiart1ev1aaatCvAUfKttLearuWrP9MDH5MBPbIqV92AaeXatLxBI9gBaebbnrfifHhDYfgasaacH8akY=wiFfYdH8Gipec8Eeeu0xXdbba9frFj0=OqFfea0dXdd9vqai=hGuQ8kuc9pgc9s8qqaq=dirpe0xb9q8qiLsFr0=vr0=vr0dc8meaabaqaciaacaGaaeqabaqabeGadaaakeaacuWG4baEgaqeaaaa@2E3D@)(*y *- y¯
 MathType@MTEF@5@5@+=feaafiart1ev1aaatCvAUfKttLearuWrP9MDH5MBPbIqV92AaeXatLxBI9gBaebbnrfifHhDYfgasaacH8akY=wiFfYdH8Gipec8Eeeu0xXdbba9frFj0=OqFfea0dXdd9vqai=hGuQ8kuc9pgc9s8qqaq=dirpe0xb9q8qiLsFr0=vr0=vr0dc8meaabaqaciaacaGaaeqabaqabeGadaaakeaacuWG5bqEgaqeaaaa@2E3F@)σx−1σy−1
 MathType@MTEF@5@5@+=feaafiart1ev1aaatCvAUfKttLearuWrP9MDH5MBPbIqV92AaeXatLxBI9gBaebbnrfifHhDYfgasaacH8akY=wiFfYdH8Gipec8Eeeu0xXdbba9frFj0=OqFfea0dXdd9vqai=hGuQ8kuc9pgc9s8qqaq=dirpe0xb9q8qiLsFr0=vr0=vr0dc8meaabaqaciaacaGaaeqabaqabeGadaaakeaaiiGacqWFdpWCdaqhaaWcbaGaemiEaGhabaGaeyOeI0IaeGymaedaaOGae83Wdm3aa0baaSqaaiabdMha5bqaaiabgkHiTiabigdaXaaaaaa@3746@. Pre-processing of the functional data was implemented as in Ref. [6]: right-handed human subjects were studied using a Siemens-Trio 3.0 Tesla imaging system using a birdcage radio-frequency head coil. Blood oxygenation level-dependent single-shot echo-planar T2-weighted imaging was obtained using scan repeat time of 3000 ms, echo time of 30 ms, flip angle 90°, and field 256 mm. The data were preprocessed for motion correction and spatial smoothing using the FSL package [19]. The link density maps were computed based on the degree of each node, and were then, for the purpose of comparison, normalized to the anatomy of the subject using the registration tool in FSL.

## Competing interests

It must be noted that G.A.C. and A.R.R. are employees of IBM Corp., whose Blue Gene supercomputer was utilized to claim computational tractability of the method.

## Authors' contributions

GAC worked on problem formulation, solution design and implementation, ARR worked on solution design and implementation, MVC worked on data collection, MB worked on data collection and experimental design, AVA worked on experimental design and problem formulation, DRC worked experimental design, problem formulation and solution design. All authors read and approved the final manuscript.
